# What do mathematics lessons look like? Analyses of primary students’ drawings

**DOI:** 10.3389/fpsyg.2023.1019299

**Published:** 2023-07-13

**Authors:** Benjamin Rott, Laura Barton, Vesife Hatisaru

**Affiliations:** ^1^University of Cologne, Cologne, Germany; ^2^Gemeinschaftsgrundschule Irisweg, Primary School, Cologne, Germany; ^3^Edith Cowan University and University of Tasmania, Joondalup, WA, Australia

**Keywords:** drawings, draw a mathematics classroom, mathematics lessons, primary students, beliefs

## Abstract

The use of student drawings to assess their experiences and beliefs about teaching and learning of mathematics has become almost a regular research method – especially when working with young students who may not express themselves well, for example, in self-report questionnaires. These methods, nevertheless, need to be improved regarding their objectivity and validity. By building on the existing research, in this study, we focus on objectivity and validity issues in drawing-based methods. We use a drawing-based instrument: *Draw A Mathematics Classroom* (DAMC) and present 104 fourth-grade students to draw a picture of their regular mathematics lessons. We especially aim to develop and validate a data coding manual with low-inferent categories; that is, well-operationalizable categories that can be used with high interrater-reliability like the presence of teachers, the arrangement of student desks, and teacher-student interactions. The results reveal that almost half of the participating students perceive their lessons as teacher-centered. The results also confirm the reliability and validity of the methodological approach. For example, in pictures where the teacher is depicted larger than the students, the teacher is also depicted in the center, and students are pictured working alone. Classroom observations support students’ perceptions, and all these show that the manual used in this study is useful to getting insights into young students’ perceptions of their mathematics classroom.

## 1. Introduction

There are many factors that influence the learning gains of students in mathematics classrooms in addition to students’ abilities. One of these factors—that is the focus of the present study—is the students’ experiences and beliefs regarding the teaching and learning of mathematics (e.g., Schoenfeld, 1989; Mapolelo, 2009; Sullivan, 2011) as students’ learning largely depends on their interactions with the teacher over learning objectives (Ball and Forzani, 2011). For example, whether doing mathematics is a singular versus group activity, or whether such learning environments are dominated by teacher instructions versus students’ explorations—such activities and interactions shape learners’ experiences and their beliefs about mathematics.

Experiences and beliefs regarding the teaching and learning of mathematics are shaped early in students’ careers. Therefore, it is important that research in this field addresses primary grades. Although there are established methods to assess beliefs, “there is considerable scope for the development of new methods and the wider use of established methods for qualitative studies” (Fraser, 2014, p. 116). We endeavor to contribute to the methodological discussions by exploring and validating the research method of interpreting students’ drawings, as common methods and instruments used to investigate experiences and beliefs—like interviews and self-report questionnaires—are less suitable for children who often struggle with understanding interview questions or questionnaire items and are not yet able to reflect upon their experiences and beliefs (cf. Rolka and Halverscheid, 2011; Döring and Bortz, 2016). Additionally, interviews are unlikely to be useful to conduct data from large samples (Döring and Bortz, 2016) and self-report questionnaires suffer from validity problems (Safrudiannur and Rott, 2020).

Our research goal is to better understand the method of analyzing and interpreting primary students’ drawings in the context of their experiences and beliefs regarding teaching and learning of mathematics. Specifically, we use self-drawn pictures of primary students (grade 4). Compared to more commonly used research methods, the coding and interpretation of drawings still needs methodological development. Therefore, in addition to the results of our study regarding the students’ beliefs, we especially focus on validating the methodological approach.

## 2. Theoretical background

### 2.1. Students’ experiences and beliefs

To date, certain student affect outcomes including students’ attitudes, feelings, or beliefs relating to mathematics, and their views about mathematicians have been widely investigated (e.g., Picker and Berry, 2000; Rock and Show, 2000; Dahlgren and Sumpter, 2010; Aguilar et al., 2016; Hatisaru, 2020a). Yet, the research in this area lacks information on the perceptions of students relating to their mathematics teaching and learning experiences. Large-scale assessments such as Trends in International Mathematics and Science Study (TIMSS) and Program for International Student Assessment (PISA) have identified important aspects of perceived school and classroom experiences of both students and teachers. Some researchers have used the TIMSS (e.g., O’Dwyer et al., 2015) or PISA (e.g., Echazarra et al., 2016) data to examine perceived teaching and learning practices in mathematics classrooms, and how particular teaching practices are related to student performance. The findings from these studies, however, are limited to responses obtained from questionnaire items as they did not include observational and/or interview data (Vieluf et al., 2012). Research shows that the statements used in questionnaires are not necessarily understood by young students in the way researchers mean (Bragg, 2007). More research and especially alternative research methods are needed not only exploring perceived mathematical experiences in the classroom but also to fill some of gaps in the existing literature.

Doing research on students’ beliefs and experiences regarding teaching and learning of mathematics is important, as such beliefs may affect the learning of mathematics. For example, students who mostly know routine exercises from their mathematics classes will most likely stop working on non-routine problems after just a few minutes and wait for the teacher to present them the “correct procedure” (cf. Schoenfeld, 1992, p. 358f.). More generally, teacher-centered approaches or lecture-style teaching, as well as emphasizing the repetition of problems and correct use of procedures, can negatively impact students’ attitudes (Hasni and Potvin, 2015), making it difficult for students to remain engaged and be successful in STEM (science, technology, engineering, mathematics) subjects (Cooper and Carter, 2016) including mathematics.

As mentioned earlier, there is an abundance of research on students’ beliefs; however, most common methodologies rely on (closed) questionnaires or interviews, which can lack validity and are less suitable for primary students (see Section 1). Therefore, we utilize *drawings* as the research tool and explore the validity and reliability of using them to address beliefs. Additionally, as a research tool, student drawings have been less used in the field of mathematics education in Germany, and we were interested in providing additional evidence with respect to students’ perceptions of the teaching and learning of mathematics in their classroom. The results contain valuable insights into the classroom mathematical practices in Germany and beyond.

### 2.2. Learner- vs. teacher-centered learning environments

Learning environments can be characterized regarding different dimensions, one of which being learner- vs. teacher-centeredness, ranging from very open project- and problem-based environments to environments in which the teachers almost always step in and do the work for the students (Hiebert and Stigler, 2004; Dole et al., 2016). The discussion about such learning environments is similar to the debate between psychologists Bruner and Ausubel who favored discovery and receptive learning, respectively (e.g., Legge and Harari, 2000).

*Per se*, none of the two teaching styles is “better” or “more effective” (by any definition of these terms) then the other. However, research has shown that some teaching practices often associated with learner-centered environments, are more favorable than others in leading to desirable learning outcomes, for example having mathematical communications, varying teaching approaches, and involving non-routine problems (Anthony and Walshaw, 2009; Bobis et al., 2011; National Council of Teachers of Mathematics [NCTM], 2014). On the one hand, compared to learner-centered environments, teacher-centered environments often rely on direct knowledge transfer, resulting in students to prefer surface instead of deep approaches to learning (Trigwell et al., 1999). On the other hand, student-centered learning can improve students’ achievement (Parker and Gerber, 2000; Hatisaru and Kucukturan, 2011; Baeten et al., 2013), motivation (Baeten et al., 2013), and attitudes (Parker and Gerber, 2000; Erdemir, 2009). It is more often related to a constructivist approach of teaching in which students are enabled to construct their own information and the teacher’s role is that of a facilitator rather than an instructor (Aktas, 2010). Learner centeredness can help students to develop their inquiry and collaboration (Hatisaru, 2014) and flexible understandings and lifelong learning skills (Hmelo-Silver, 2004).

It is noteworthy that (1) In this article, we do not intend to compare the effectiveness of different teaching approaches and learning environments and will not measure learning outcomes. (2) Some, or even most of the mentioned qualities associated with learner centeredness do not directly result from the role of the teacher, but from the use of tasks that foster cognitive activation, from student collaboration, etc. However, empirical research has shown that such qualities often are related to the role of the teacher. Thus, it is a simplification to speak of teacher vs. learner centeredness, but a reasonable one. Additionally, it is much easier to observe the role of the teacher than to assess the quality of tasks, group works, etc.—especially for young children who cannot reflect upon such qualities. Therefore, we aim to explore whether we can access students’ experiences and beliefs regarding such environments via their drawings by focusing on the role of the teacher.

### 2.3. Drawing as a research tool

Young children start to draw from infancy and many of them continue to draw because it is enjoyable, it produces beautiful representations, and it allows them to express feelings, emotions, and ideas that words alone cannot describe.

Drawing is independent of language-based methods and is non-textual, hence as a research method, it can provide researchers with an alternative and versatile way of knowing (Pehkonen et al., 2016; Hatisaru, 2022). Researchers use drawing and/or multimodal research methods (e.g., drawing; text; or verbal responses) to explore participants’ understandings of different phenomena. Thus, drawing has become an important tool for researchers interested in image-based research methods. Literat (2013) argues that its lack of dependence upon linguistic proficiency makes drawing particularly suited for working with children, and others argue that it helps bridge the gap between children and adults (Søndergaard and Reventlow, 2019).

In this research, we are interested in exploring how school children experience mathematics classrooms and especially if analyzing their drawings is a valid method to assess their experiences and beliefs. We have drawn upon previous work in mathematics and science education to help us to address the perceived experiences of them on how mathematics is taught in their classrooms.

Drawings have been found to be valid indicators of classroom experiences (Gulek, 1999) and have the potential to provide rich and valid information (e.g., Laine et al., 2020). They allow school or classroom environments to be documented from the perspective of students, “the most assiduous observers of school and classroom life” (Haney et al., 2004, p. 243). Drawings “can provide a valuable catalyst to document, change, and improve what goes on in schools” (Haney et al., 2004, p. 243). For some time, therefore, drawings have been used to evaluate classroom teaching and learning in school subjects including mathematics (e.g., Pehkonen et al., 2016).

For decades now, the “Draw A Mathematician Test” (DAMT) (Picker and Berry, 2000)—which had been adopted from Chambers’s (1983) “Draw A Scientist Test”—as well as variations of the test have been widely used to elicit data from students about their perceptions of mathematics (e.g., Rock and Show, 2000), mathematics and mathematics education with a focus on motivation (e.g., Dahlgren and Sumpter, 2010), mathematicians (e.g., Picker and Berry, 2000; Aguilar et al., 2016; Hatisaru, 2020a), and mathematics teaching (e.g., Pehkonen et al., 2011; Hatisaru, 2020a).

In the following section, we present perceived experiences for teaching and learning of mathematics found in student drawings.

### 2.4. Previous research regarding drawings

In one of the ground-breaking studies in this field, Picker and Berry (2000) investigated the perceptions of mathematicians held by lower secondary school students (12 to 13 years old) in the United States, the United Kingdom, Finland, Sweden, and Romania by using the DAMT, and compared students’ images in these countries. With small cultural differences, certain stereotypical images of mathematicians were found to be common among students. Mathematicians quite often were pictured as people having special powers, and sometimes as foolish people. As also found in Rock and Show (2000), many students seemed to believe that mathematicians do the same work as students do in their own mathematics classes such as arithmetical computations, area and perimeter, and measurement. Mathematicians and their work were invisible for those students. According to Picker and Berry (2000), school-related factors such as often experiencing direct teaching methods through which students do not see the applications of mathematics enough, is one of the sources of students’ images of mathematicians.

In another study in the United States, most students’ drawings of mathematicians were shown in the classroom. Young respondents (kindergarten—grade 8) named tools they were familiar with from their own classrooms (e.g., paper, pencils, whiteboards, etc.) as tools of mathematicians, second and third grade respondents mentioned calculators, rulers, geometric shapes, while fourth grade and middle school students expanded their responses to include computers, calculators, and protractors (Rock and Show, 2000).

Pehkonen et al. (2011) used drawings to reveal young students’ (8–9 years old) conceptions on mathematics and its teaching in Finland. Among 153 student drawings, every second drawing included indications to attitudes towards mathematics such as mathematics is nice, easy, dull, or difficult. As opposed to findings in Picker and Berry (2000), no negative views about the teacher were found in student drawings. The depicted mathematics lessons contained many activities. Two thirds of the participating students pictured a classroom environment where students in the picture were in action such as thinking, speaking, or discussing. Laine et al. (2013) further analyzed these drawings to study the kind of emotional atmosphere in a mathematics lesson that could be seen in students’ depictions. Mostly a positive emotional atmosphere was found in the pictures. Pehkonen et al. (2016) were curious about what could be found in the same drawings relating to mathematics teaching, and they examined the drawings to find out the types of work experienced in mathematics lessons through the eyes of students. The most frequent work experienced in students’ pictures was found to be ‘Independent work’ (students work individually for solving problems at textbooks or given by the teacher) and ‘Work with the teacher in charge’ (the teacher teaches the whole class, or all students work on the same task). ‘Group work’ (students work with classmates on a task) was less common. In this set of research studies, Pehkonen and his colleagues found drawings as an efficient way of collecting data to explore students’ experiences in mathematics lessons and offered the drawing tasks to practitioners as a possible way to obtain and evaluate information about students’ perceptions pertaining to mathematical experiences.

In Spain, Remesal (2009) used drawings to explore how primary school students (7 to 8 years old) perceived assessment practices in the classroom, and how students’ conceptions might be shaped by their actual classroom experiences. In a case study design, two practicing teachers and their twelve students (six from each teacher’s class) participated in the research. Data were collected through semi-structured interviews with teachers and their students, classroom observations, artefacts used in the assessment of mathematics learning, and students’ drawings of mathematics classrooms. Remesal (2009) reported that:

The main common result of these cases is the identification of young primary pupils’ capability of perceiving assessment practices as ruled by distinctive norms and conventions in the classroom among other classroom routines: ‘someone is to ask and someone is to respond,’ ‘someone is to show the work and someone is to mark the work,’ ‘grades are given and the parents are informed.’ This awareness develops even though the teachers themselves might not believe 8-year-olds are capable of such insights (p. 47).

In her study investigating the image of mathematics held by a large group of middle school students in Turkey through examining students’ drawings, Hatisaru (2020a) found that students associated mathematics narrowly with only numbers and arithmetic, and the work of mathematicians with solving textbook questions or performing calculations. Some of the students depicted great mathematicians in the past (e.g., John Nash, Ali Qushji, or Pythagoras), and they thought that the main activity of even those mathematicians is studying to solve algebra, numbers, or geometry practice questions (Hatisaru, 2020a). A further investigation into the same student drawings (Hatisaru, 2019) revealed that in the drawings the most common mode of instruction was highly teacher-directed. No evidence of group work or student-oriented mode of instruction existed. A whiteboard and/or books were the most observed teaching resources in classroom portrayals. Technological tools appeared the least often in these drawings. An important part of the teacher’s activity in the classroom were lecturing, explaining, solving exercises, and disciplining. When present, students’ desks were in orderly rows. The interactions among students and between the teacher and the students were limited.

Associating mathematics predominantly with calculations or operations was also evident in another study implemented in Turkey. In that study, Ucar et al. (2010) implemented interviews and used drawings to investigate the beliefs about mathematics and mathematicians of nineteen elementary school students (grades 6 to 8) attending a supplementary school, where students are instructed out of their school times. Both student interview responses and pictures revealed that students viewed mathematics as numbers, formulas or computations, and believed that mathematicians could be needed, for instance in the industry, for their computational skills. To students, being good at mathematics meant finding a correct answer to questions quickly. For several students, in mathematics learning, finding a correct answer to a question was sufficient, understanding the question was not that important.

In her research exploring drawings of learning in the classroom depicted by a group of 6- to 7 year-old students in a primary school in the United Kingdom, Lodge (2007) stated that when children draw, they make decisions and choices about what to include or not in the drawing. The main sources of their decisions might be affected by cumulative cultural text, the current or past experiences, and individual preferences. The author found that all these three sources were seen in the students’ drawings analyzed. However, as the students’ drawings showed some disparity, she made a warn against the assumption that we ‘know’ how a young child views their learning, or that children in the same class share ‘a common view’ of learning.

Aktas (2010) analyzed drawings and semi-structured interviews of 41 fourth-grade students from a Turkish school. In that study, the vast majority of students depicted their teacher as an “instructor-informant” inferring that most students see themselves as passive receivers. The author concludes that the intended change of the Turkish curriculum towards more student-centered and constructivist learning environments has not been successful at the time of the study.

Kanyal and Cooper (2010) compared drawings, as well as interviews, and photographs of 12 children from England and 15 children from India being asked about their “actual” and their “ideal” school experience. Students liked being with their friends in school, but wanted to spend more time outside in both countries.

In a 2 year study, Streelasky (2017) observed 35 Canadian primary students’ perceptions of their learning experiences. Several methods were used, including interviews, group discussions, photographs, and drawings. A major finding was the importance of the outdoors, that is outside activities and a high value placed on peers as well as other living things such as animals.

Together, the reviewed literature has shown that drawings of students contain rich information on their thoughts about teaching and learning of mathematics, and sometimes about what is happening in the classroom. However, identifying students’ beliefs in their drawings “is related to a large amount of subjectivity in interpretation and will certainly not allow for an unambiguous classification” (Rolka and Halverscheid, 2011, p. 522), which is why we are going to thoroughly analyze this method.

### 2.5. Criticizing the analyses of students’ drawings

In many studies that analyze students’ drawings, this method is either taken for granted—that is not reflected upon and questioned regarding its validity—, and/or used in conjunction with other data—most often interviews. Both could imply that the method has some unexplored flaws or is not seen as a reliable and valid method that can be used on its own. In this study, therefore, we want to explore whether the analysis of drawings can be used on its own in a valid way. But first, we shortly discuss possible flaws of interpreting students’ drawings.

Even though “[p]upils’ drawings seem to be a powerful method to gather information from small children” (Pehkonen et al., 2016, p. 167), gathering such information relies on interpretations of the drawings. For example, Pehkonen et al. (2016) used the “[facial expressions of] pupils’ and teacher’s faces in drawings […] to conclude how the pupil who did the drawing has experienced the emotional atmosphere in class” (p. 172). They used two reviewers who reexamined and discussed their classifications when they did not agree (Pehkonen et al., 2016, p. 173). But still, those classifications rely—amongst others—on the children’s abilities to depict facial expressions in such a way that they can be interpreted validly and on the reviewers’ interpretations. Especially the latter makes such a coding high-inferent, that is depending on interpretations, but not on operationalizations, or on countable or measurable objects.

Another example is the study of Rolka and Halverscheid (2011), in which fifth graders’ drawings were coded for the mathematical world views by Ernest (1989): the instrumentalist, the problem-solving, and the Platonist view. Even though the authors report interrater-reliability scores (Cohen’s kappa) between 0.21 and 0.58, such codes still imply a lot of interpretation, especially keeping in mind that fifth graders might not fully comprehend the philosophical background of the world views.

Finally, we discuss the study by Gulek (1999) in which “a rating from 1 to 4 was assigned to each drawing for each of the two classroom constructs/traits” (p. 44). These four ratings are 1: Highly, and 2: Moderately teacher-directed mode of instruction, as well as 3: Moderately, and 4: Highly student-centered mode of instruction. For each of these ratings, Gulek developed indicators to help him decide which rating to apply to a drawing. For example, in a highly teacher-directed classroom, “only the teacher [is] depicted, students are not present in the picture” or “if depicted, student desks are in rows” (p. 124). Such indicators make it easier to code with sufficient interrater agreement, yet, still, there is interpretation in some codes, for example whether “teacher talk, if any, is lecturing or disciplining” (Gulek, 1999). Also, decisions like whether a classroom is highly or moderately teacher-directed depend on the fact whether students are depicted in the drawing or not. However, it is easily imaginable to have a drawing of a highly teacher-centered classroom in which students are depicted.

In the present study, we address the mentioned problem with high-inferent codes that heavily rely on interpretations by suggesting a coding manual with highly operationalized coding instructions, resulting in a low-inferent coding.

## 3. Methodology

### 3.1. Participants

We collected data in four 4th-grade classes (coded as A–D to assign students’ drawings to the classes; that is, Class A, Class B, etc.) from two primary schools in central Germany. In total, 104 students (9–10 years old) participated in the study. The research was approved by the ethics committee of the relevant university, and prior to collecting data, the teachers, school principals, and parents in participating schools gave written consent for the study. Data collection took place in February and March of 2020. In Germany, first contact restrictions because of the COVID-19 pandemic had been introduced in mid-March—at this point, the restrictions were mostly self-imposed with no “official lockdown” being implemented, yet. The second author had already collected data in three of the four classes (one in February, two in March) before restrictions were initiated. In the fourth class, Class A, the drawings were collected by the teacher shortly after restrictions had been implemented, because the researcher was not allowed to enter the school anymore. At that time, restrictions applied only to people outside of the school; there were no restrictions regarding student interactions, group work, etc., yet.

The curriculum for 4th-grade students in the respective federal state of Germany defines (i) process- and (ii) content-related competencies. The process-related competencies are (i.1) problem solving/being creative, (i.2) modeling, (i.3) reasoning, and (i.4) representing/communicating; the contents, which should be taught, are organized into (ii.1) numbers and operations, (ii.2) space and form, (ii.3) size and measure, and (ii.4) data, frequencies, and probabilities (MSW, 2008).

### 3.2. Methods

To assess our students’ experiences and beliefs, we used a variation of the DAMT (Picker and Berry, 2000) (please see Appendix A) with the following instruction: *Draw your mathematics lessons with your teacher. The picture should show what you know about his or her work*. (German original: *Male deinen Mathematikunterricht mit deiner Lehrerin/deinem Lehrer. Das Bild soll zeigen, was du über sie und ihre Arbeit weißt.*)

We chose this instruction to draw the students’ attention to a usual classroom environment. This way, we intended to get insight into the environments that shape their experiences regarding mathematics lessons.

Students’ pictures were scanned and converted into a PDF file. The first two authors then independently coded all pictures according to the manual that is described below. Interrater agreement was good at Cohen’s Kappa = 0.88, cases of disagreement in coding individual drawings have been discussed and then recoded consensually.

In addition to collecting the pictures, the second author (a) spoke with the children about their drawings and (b) observed teaching practices in the participating classes.

(a) The second author had 10% of the children explain their pictures to check whether our interpretations of depicted persons and objects were correct, which they were in all cases. She also asked the children how they proceeded in drawing their pictures and what was important to them. The children’s answers regarding their classroom experiences were all in line with the observations, confirming, for example, whether teaching was mostly organized in teacher–student conversations or in small-group work, or whether teachers focused on arithmetic compared other topics like geometry or data and chances.

(b) For 12 weeks, the second author visited three of the four classes (all but Class A) and took notes on teachers’ and students’ classroom routines. On the basis of these observations and notes, the classroom environments were interpreted with regard to the distinction between a teacher- or student-centered environment. Of note, in one of the classes, guests were not allowed because of restrictions in the course of the COVID-19 pandemic.

### 3.3. Coding the pictures

Aiming to improve the existing coding schemes for students’ drawings from the literature (see Section 2.5), we developed a coding scheme focusing on aspects that are operationalizable, for example whether students are depicted or what mathematical content is shown. The process of developing the categories of the manual can be described as an application of Qualitative Content Analysis (Mayring, 2000), using a deductive-inductive approach. Deductively, we took inspiration from existing coding schemes (especially Gulek, 1999 and Hatisaru, 2020b); inductively, we recorded every detail of the drawings that seemed to be interesting but were not covered by categories, yet.

The full coding manual is given in Appendix B, examples and explanations are given in the section “Data Analysis and Results” (Section 4).

## 4. Data analysis and results

To give readers an impression of our data, we start with describing and showing typical students’ drawings (Section 4.1). We then evaluate the coded pictures more thoroughly (Section 4.2), and finally focus on analyzing the data with regard to validity and reliability (Section 4.3). Please note, we aim at using well operationalized codes, not holistic interpretations of the pictures. Therefore, we show several pictures, focusing on observable details.

### 4.1. First impressions

**Figure 1:**
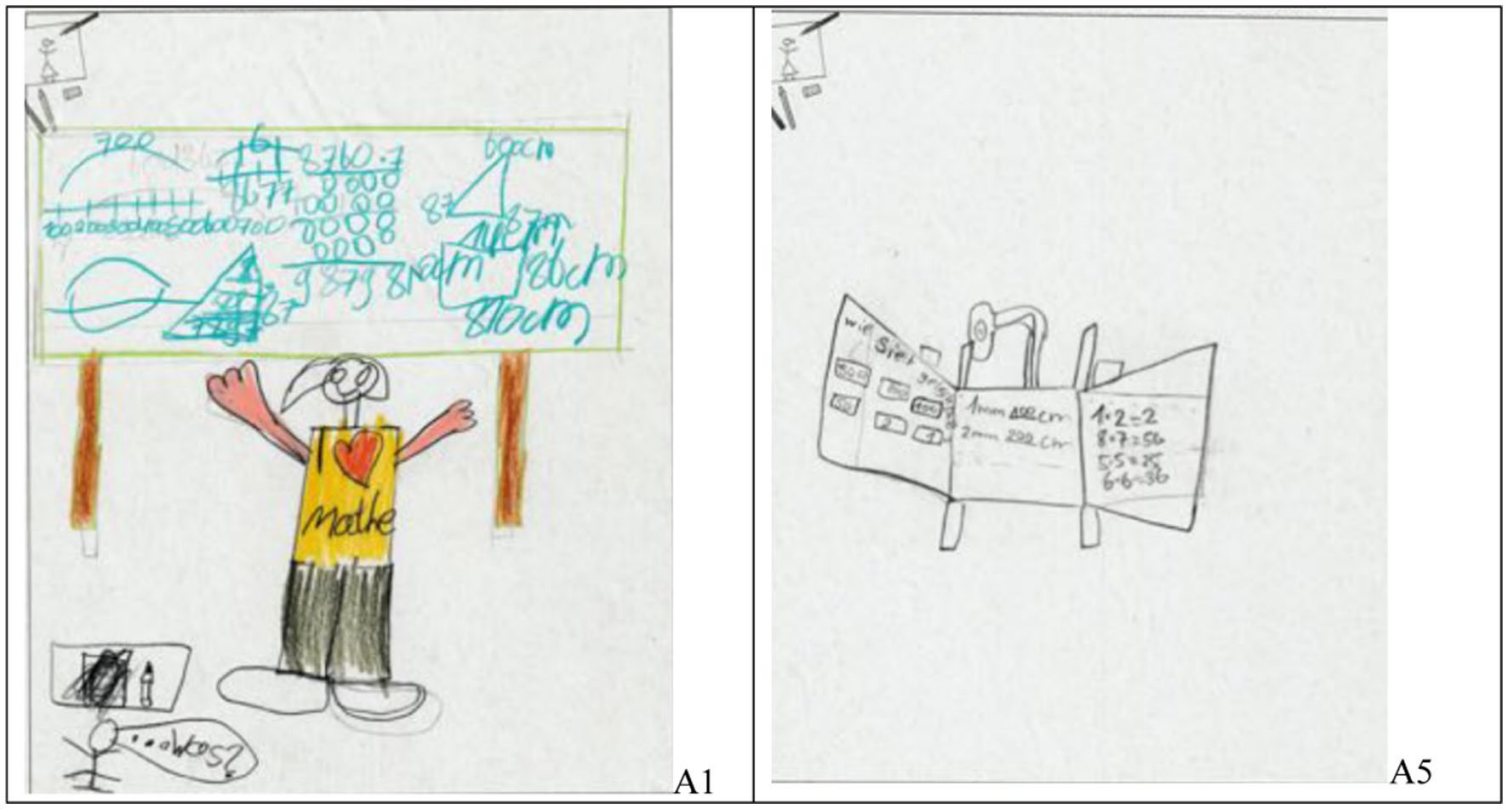
Examples of students’ drawings with a board at the center and with (left, student A1) or without persons (right, student A5).

Looking at the students’ pictures, we often see the board (i.e., blackboard, whiteboard, or smartboard) in the center, sometimes with and sometimes without persons (see Figure 1).

**Figure 2:**
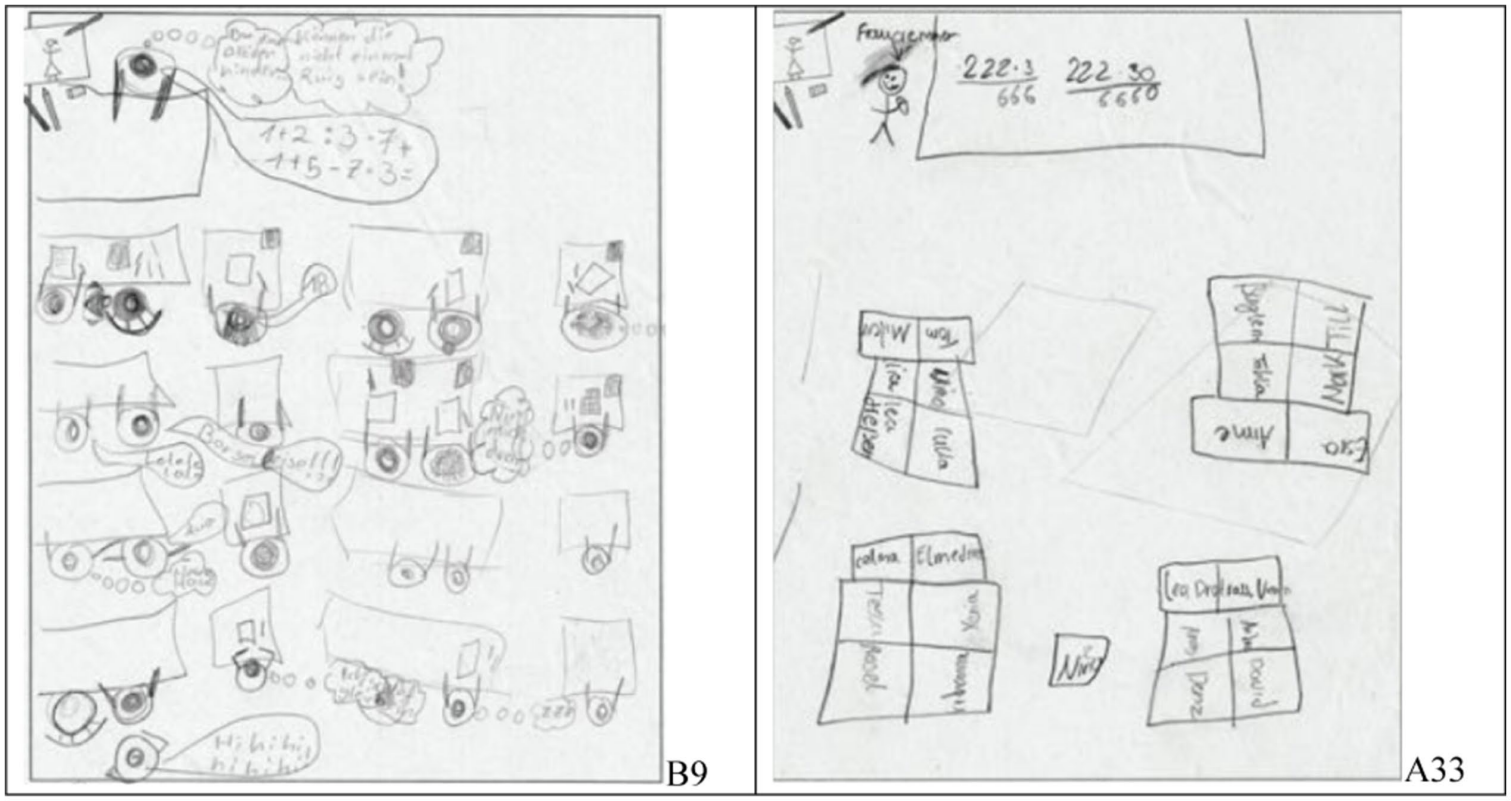
Examples of students’ drawings with students drawn and seated in rows (left, student B9) or written and seated in clusters (right, student A33).

There are pictures with students seated in rows as well as seated in clusters (see Figure 2).

**Figure 3:**
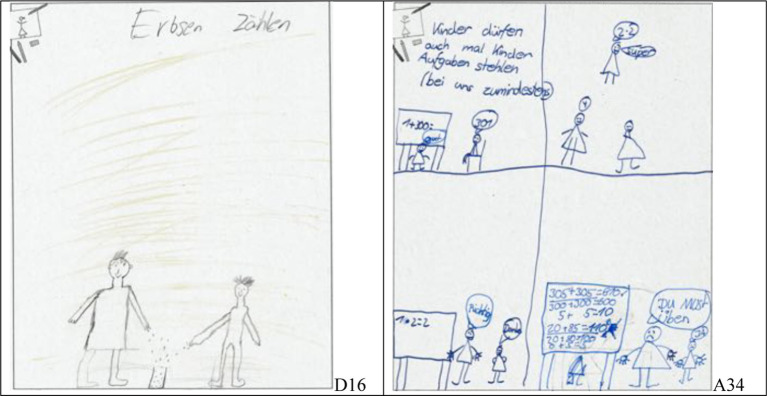
Examples of students’ drawings; students shown in an activity named “Erbsen zählen” which translates to “counting peas” (left, student D16) and a “comic strip” (right, student A34).

Most pictures show arithmetic tasks (see Figures 1, 2), but others depict specific actions like “counting peas” (Figure 3, left). A few papers do not show a single classroom situation but several in a “comic strip”-like way (Figure 3, right).

### 4.2. Coding the pictures

In coding the pictures, we tried to get a better overview of what is depicted in the students’ drawings, make them comparable, their attributes countable, and get insight into the students’ experiences and beliefs. We present our findings under the themes “teachers” (Section 4.2.1), “students” (Section 4.2.2), and “classes” (Section 4.2.3).

#### 4.2.1. Teachers

**Figure 4:**
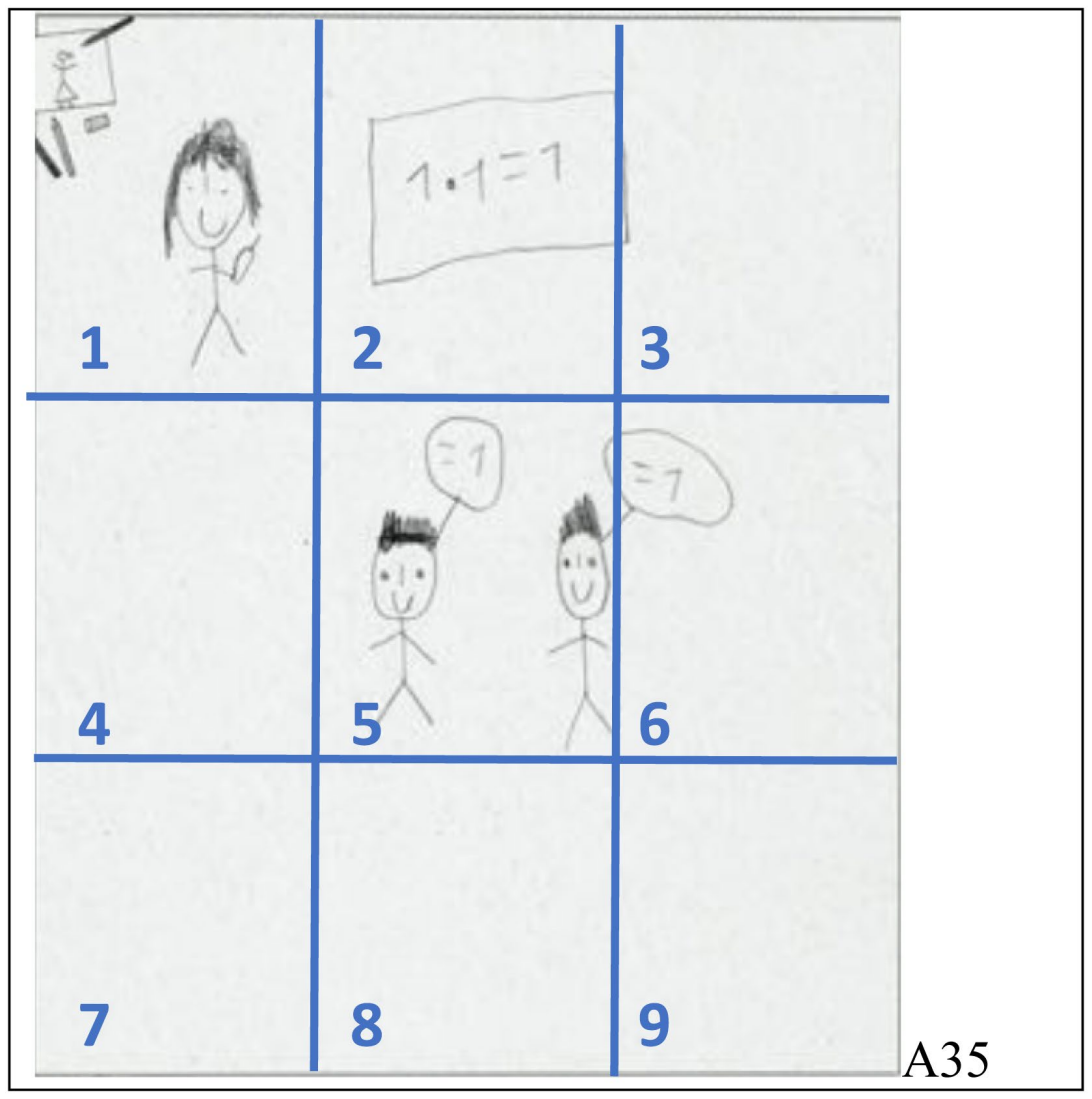
Determining the position of drawn persons and objects (like the teacher) by dividing the drawings equally into 9 areas (student A35).

The first thing we did was looking at the teachers as they are most often positioned prominently in the drawings. More specifically, we (1) looked at the teacher’s size compared to their students’ sizes and (2) at the teacher’s position within the pictures. Regarding analysis (1), taking into account that adults are taller than children, teachers can be depicted as bigger (e.g., Figure 1, left), about the same size (e.g., Figure 2, left), or smaller than the students. When no teachers or students are shown, “no comparison” is possible. Regarding analysis (2), we divided the pictures equally into 9 areas (see Figure 4). When a teacher was mostly shown in area 5 (could extend into other areas), we coded “center”; we coded “left” or “right” for the areas 1, 4, and/or 7 or 3, 6, and/or 9, respectively (even though we allow for the upper and lower corners, most “left” or “right” teachers are shown in areas 4 or 6, respectively); finally, we coded “upper or lower edge” for the areas 2 or 8. Results of this coding are shown in Table 1.

**Table 1:** Teachers’ sizes and positions in the students’ drawings.

(1) Teacher size (compared to students)			(2) Teacher’s position		
Smaller	2	1.9%	Centre	19	18.3%
Same	23	22.1%	Left	25	24.0%
Bigger	45	43.3%	Right	32	30.8%
No comparison	34	32.7%	Upper or lower edge	10	9.6%
			Comics	7	6.7%
			No teacher	11	10.6%
Sum	104	100%	Sum	104	100%

The relative size of the teachers (considering that teachers as adults are larger than children) is most often bigger than the students’ size, twice as often as equal size; and the teachers are almost never depicted smaller than the students. The teacher is often positioned in the middle row (left, center, or right) of the picture (73.1%, or 88.4% if you do not count pictures without teachers and comics). Both—position and size—indicate that the teachers have a very important, maybe even dominant role in the depicted classrooms as also inferred by Picker and Berry (2000).

#### 4.2.2. Students

Next, we examined the depicted students, more specifically (3) their position in the classroom and (4) the arrangement of their desks. Regarding analysis (3), we coded whether they were shown at their places or somewhere else in the classroom (e.g., at the board or in the center of the depicted room). Regarding analysis (4), the desks, we identified whether none or only a single desk is drawn or if there are multiple desks, whether they are depicted in rows or clustered for group work. Findings were mostly in consistent with the literature (e.g., Dahlgren and Sumpter, 2010; Pehkonen et al., 2016; Hatisaru, 2019, 2020b).

In Table 2, we see that more than 40% of the pictures do not include students, which is at least to some degree surprising, as the pictures were drawn from students’ perspectives.^1^ Most of the pictures with students show them at their places, ready to write and/or work. Only less than 9% of the pictures show arrangements of the desks that are suited for group work; most pictures show students sitting alone at their desks (like observed in, e.g., Pehkonen et al., 2016 and Hatisaru, 2019).

**Table 2:** Students’ position in the classroom and the arrangement of their desks.

(3) Students’ position			(4) Students’ desks		
At their place	38	36.5%	Single	22	21.2%
Not at their place	17	16.4%	Rows	24	23.1%
Both (multiple s.)	4	3.9%	Clustered	9	8.7%
No students depicted	45	43.3%	None	49	47.1%
Sum	104	100%	Sum	104	100%

In addition to the position of the students and their desks, we decided to code some additional aspects in the drawings to give a complete picture of students’ perceived experiences and beliefs. For example, we coded: (5) the students’ activities—working alone, working in groups, or other (e.g., talking to the teacher, walking through the classroom)—and (6) the conversation between the teacher and students, giving particular attention to who is shown speaking (“not applicable” is coded when there are neither teachers, nor students depicted).

The data in Table 3 indicates that students are often shown at their place or desk and working alone (about 60% in both cases if pictures without students are not counted). This indicates that students do not work often in groups but mostly alone which is supported by the depicted activities as no group-based, open tasks are shown (see below). This impression is supported by the arrangements of the students’ desks (see Table 2). Also, there are only very few pictures in which only students speak, and even fewer in which students speak with other students.

**Table 3:** Students’ activities and teacher–student conversation.

(5) Students’ activities			(6) Teacher–student conversation		
Working alone	35	33.7%	Only teacher	14	13.5%
Working in group	2	1.9%	Only students	5	4.8%
Other	22	21.2%	Teacher and students	16	15.4%
No students	45	43.4%	No conversation	66	63.5%
			Not applicable	3	2.9%
Sum	104	100%	Sum	104	100%

We also (7) analyzed the mathematical tasks that are shown on the black-, white-, or smartboards and (8) the representation modes (according to Bruner, 1966) which are shown in the pictures (distinguishing between the enactive, iconic, and symbolic modes as well as combinations of those modes). The results are presented in Table 4.

**Table 4:** Mathematical activity and representation modes of relevant activities.

(7) Mathematical tasks (on the board)			(8) Representation mode		
Simple arithmetic	82	78.8%	Enactive (E)	4	3.8%
S.A. and lengths	2	1.9%	Iconic (I)	1	1.0%
S.A. and geometry	3	2.9%	Symbolic (S)	85	81.7%
Geometry	3	2.9%	EIS	1	1.0%
None	10	9.6%	IS	3	2.9%
Undecidable	4	3.8%	None	10	9.6%
Sum	104	100%	Sum	104	100%

Being asked to draw a usual mathematics lesson, the majority of the students depicted simple arithmetic in a symbolic form (i.e., formulas, equations) as the content of the lesson as also observed in Hatisaru (2020b).

Finally, we recorded the (technological) tools drawn by the students. Only 4 out of the 104 pictures show a computer in the classroom, even though three of the four classrooms had a computer in them, whilst only one of those computers had been used during the time in which the classes were observed by the second author. In one picture, an overhead projector was seen. Four pictures show compass, ruler, and set square in a size that fits the board; and another two pictures show a pointing stick for the teacher, even though no classroom had such a stick. This shows, sometimes students show preference to what to include or not include in their pictures (Lodge, 2007). Regarding the board, six pictures do not show any board, nine show a smartboard (with a visible projector attached to it) and the other 89 pictures show a black- or whiteboard (without visible technology attached).

All codes presented are deliberately low-inferent, meaning that they are well-operationalized and easy to code with high interrater agreement. We are now going to interpret those codes with regard to their validity, that is whether we can get meaningful information regarding the students’ classroom experiences.

#### 4.2.3. Classes

In the previous sections, the results give insights into the whole group of 104 students and their experiences. In this section, we sort the pictures by the students’ classes. Taking into account previous research (see Section 2.3) and the considerations from the previous sections, we make the following assumptions regarding teacher- versus student-centered classroom experiences (see Table 5).

**Table 5:** Attributes of the students’ drawings indicating rather teacher-centered (left) or student-centered (right) learning environments.

Teacher-centered learning environments are rather associated with	Student-centered learning environments are rather associated with
Teacher bigger than students	
No students depicted	Students are depicted
Only one student depicted	More than four students are depicted
Only a single student’s desk is depicted	
Students’ desks are shown in rows	
Students are working alone	Students are working in groups
Only the teacher speaks	Only students are speaking
	Teacher and students are speaking

As the four classes were of different sizes, in Tables 6, 7, only relative numbers are given. In each row, the maximum value is marked. The data suggests that especially Class B is regularly taught in a teacher-centered way, whereas Class D is often taught in a student-centered way.

**Table 6:** Distribution of attributes that hint at teacher-centered learning environments.

Code/class	Class A (*n* = 45)	Class B (*n* = 20)	Class C (*n* = 20)	Class D (*n* = 19)
Teacher bigger than students	33.3%	45.0%	60.0%	47.4%
No students depicted	48.9%	50.0%	45.0%	26.3%
Only one student depicted	28.9%	25.0%	10.0%	21.1%
Only a single student’s desk is depicted	20.0%	35.0%	25.0%	5.3%
Students’ desks are shown in rows	4.4%	45.0%	35.0%	31.6%
Students are working alone	22.2%	30.0%	45.0%	52.6%
Only the teacher speaks	24.4%	5.0%	0.0%	10.5%

**Table 7:** Distribution of attributes that indicate student-centered learning environments.

Code/class	Class A (*n* = 45)	Class B (*n* = 20)	Class C (*n* = 20)	Class D (*n* = 19)
Students are working in groups	0.0%	0.0%	0.0%	10.5%
Students are depicted	51.1%	50.0%	55.0%	73.7%
More than four students are depicted	15.6%	15.0%	15.0%	10.5%
Only students are speaking	4.4%	5.0%	10.0%	0.0%
Teacher and students are speaking	15.6%	25.0%	5.0%	15.8%

### 4.3. Reliability and validity of the coding regarding students’ classroom experiences

In this section, we further analyze the coded data to explore its internal consistency, reliability, and validity with regard to the students’ classroom experiences. To check the internal consistency (comparable to measures like Cronbach’s alpha) and validity of the codings, we look at selected subsamples of our data.

#### 4.3.1. Individual pictures

Above, we listed the (technological) tools depicted in the students’ drawings. One of the most trustful ways to check the reliability of the drawings (and, therefore, the drawing method) is to compare the drawn tools to the real-world classrooms. For example, 9 pictures show a smartboard (a digital whiteboard) and all of those pictures have been drawn by students from class A, which is the only classroom of our sample that actually contained a smartboard. The other tools also are in line with the actual environments; thus, indicating reliable information in the drawings (e.g., Gulek, 1999; Lodge, 2007; Remesal, 2009).

#### 4.3.2. Teacher size

A majority of the teachers is depicted “bigger” than the students (in relation to normal size differences between children and adults, see above), which can be seen in Table 1. This could mean that teachers in the drawn classrooms play a more important role than the students, implying teacher-centered learning environments. To investigate this hypothesis, we analyze this subgroup of pictures with “bigger” teachers. To do so, we use the same codings as above to produce another six analyses that refer to the analyses (1)–(6) presented in Tables 1–3—now with an additional “b,” which stands for “bigger (teacher),” in the numbering.

As seen in Table 8, analysis (1b) shows the relevant numbers of drawings. In this subgroup of drawings, we see (2b) even more teachers are depicted in the center of the picture (29.0% vs. 18.3%; but about the same number in the middle row). Also (3b), more students are depicted at their place (53.3% vs. 36.5%) and (5b) working alone in this subgroup (48.9% vs. 33.7%). There are (4b) slightly larger percentages of single student’s desks and students’ desks in rows (60% vs. 44.3%; see Table 9) as well as (6b) only teacher speaking (17.8% vs. 13.5%; see Table 10); however, the latter should not easily be compared to the whole group as in the subgroup, “not applicable” is impossible as there are no pictures without persons. In summary, the data suggest that “bigger” teachers are well related to teacher-centered routines. As this is not a quantitative study, we refrain from arguing statistically at this point. However, interested readers find the results of chi-square tests that confirm these results in Tables 11–15 in Appendix C.

**Table 8:** Analysis of drawings depicting a teacher that is larger than the students: teacher’s position.

(1b) Teacher size (compared to students)			(2b) Teacher’s position		
			Centre	13	29.0%
			Left	10	22.2%
Bigger	45	43.3%	Right	13	29.0%
			Upper or lower edge	4	8.9%
			Comics	2	4.4%
Other	59	56.7 5	Other	3	6.7%
Sum	104	100%	Sum	45	100%

**Table 9:** Analysis of drawings depicting a teacher that is larger than the students: students’ positions and the positions of their desks.

(3b) Students’ position			(4b) Students’ desks		
At their place	24	53.3%	Single	13	28.9%
Not at their place	9	20.0%	Rows	14	31.1%
Both (multiple s.)	4	8.9%	Clustered	5	11.1%
No students^a^	8	17.8%	None	13	28.9%
Sum	45	100%	Sum	45	100%

a In these cases, teacher size was compared to students’ desks.

**Table 10:** Analysis of drawings depicting a teacher that is larger than the students: students’ activities and teacher–student conversation.

(5b) Students’ activities			(6b) Teacher–student conversation		
Working alone	22	48.9%	Only teacher	8	17.8%
w. in group	0	0.0%	Only students	3	6.7%
Other	15	33.3%	Teacher and students	8	17.8%
No students	8	17.8%	No conversation	26	57.8%
			Not applicable	0	
Sum	45	100%	Sum	45	100%

#### 4.3.3. Classes

We can validate the impressions from results regarding the classes via a comparison with classroom observations. In addition to the pictures, we have observation notes from the second author who visited the classes for a period of several weeks. Her impressions were:

- The teacher of Class B was originally trained to be a secondary, not a primary teacher; and he was not trained to be a mathematics teacher. He mostly used tasks from the official textbook and sometimes handed out copied tasks from another textbook. The chosen tasks were mostly closed with no room for interpretation or discussion. He structured the lessons in very small steps and gave solutions after every step; especially in reflection phases, he made sure that all students got “the one, right answer.” Often, he seemed to be uncertain about how to react to students’ questions. This way of teaching was interpreted as “teacher centered” in this study.
- The teacher of Class C was also not trained to be a mathematics teacher at primary schools, but as a special education teacher. She was also adhered to the textbook and mostly followed the textbook scripts, explanations, or examples in designing her lessons. Her teaching style was interpreted to be teacher-centered, but not as clearly as the style of teacher B as teacher C sometimes encouraged cooperative forms of learning (especially when it was recommended by the textbook script).
- The teacher of Class D used many action-oriented and cooperative forms of learning. The rhythm of her lessons was often the same: after a short introduction, the children would work independently on open-ended problems and they presented the results to each other with an open discussion following the presentations. Teacher D never highlighted her solution to be the right one; sometimes, there were so many ideas, that the discussion had to be continued in the next lesson. The students were encouraged to use research notebooks to gather their observations. In our interpretation, this teacher showed the most student-centered ways of teaching.

The data fit well to the observations with Class B showing the most maximum values in Table 6 and Class D showing the most maximum values in Table 7.

## 5. Discussion and conclusion

Analyzing students’ drawings is an emerging research method that enables insights into students’ experiences and beliefs that other methods can hardly provide. For example, compared to commonly used (self-report) questionnaires, drawings can be used even with very young students and compared to classroom observations or interviews, drawings are cost-effective as drawing-based data can be collected from a large number of students. Additionally, drawings suffer less from limitations like issues with validity and social desirability that questionnaires are faced with (Di Martino and Sabena, 2010; Safrudiannur and Rott, 2020). However, the objectivity and validity of analyzing drawings still needs to be thoroughly explored as sometimes findings are based on assumptions and interpretations by the respective researchers. In this study, we have addressed these methodological issues (See Tables 11–15 in Appendix C).

The topic of our study is observable instructional arrangements and teaching methods, namely teacher-centered vs. student-centered teaching approaches. Whilst such “sight structures” (as termed by Kunter and Voss, 2013) do not have the same power for explaining student learning progress as others such as classroom management, cognitive activation, or individual learning support (i.e., “deep structures,” Kunter and Voss, 2013), some instructional arrangements are correlated with unfavorable learning approaches. They are, therefore, important for empirical research. Previous studies have shown that this especially occurs in teacher-centered environments (Trigwell et al., 1999).

Methodologically, in this study, we asked students to draw their typical classroom environments, as their “perceptions about the role of their teachers and how they might contribute to their learning begin to be formed once they start school” (Taylor et al., 2005, p. 728). Specifically, we were interested in assessing learning environments that shape students’ experiences regarding their usual mathematics classes by making them tell—or rather show—us their perspectives of their mathematics lessons. To do so, we used a variation of the *Draw A Scientist Test* (DAST) (Chambers, 1983) and the *Draw A Mathematician Test* (DAMT) (Picker and Berry, 2001): the *Draw A Mathematics Classroom Test* (DAMC) (Hatisaru, 2020b) (see Appendix A). Such an assessment can generate rich data (see also Hatisaru, 2022); yet compared to interviews, data collected by using DAMC or alike instruments from a large number of participants can easily be generated and evaluated.

Compared to quantitative methods (like using questionnaires), the interpretation of drawings—especially when focusing on students’ beliefs—can be subjective or high-inferent (cf. Rolka and Halverscheid, 2011). We, therefore, developed a low-inferent coding manual focusing on observable characteristics of the drawings that are well-operationalizable and can be used with high interrater agreement—with codes like whether teachers or students are depicted, whether students’ desks are shown in rows or clustered, and so on. To address the validity of our codings, we compared them to classroom observations.

The results of our study—analyzing 104 drawings of 4th grade students—highlight the possibilities of the chosen research method. That is, coding drawings with the manual presented in Appendix B allows analyzing data from mid- to large-sized groups, getting insights into individual experiences as well as experiences of groups like classmates. Especially when combining the coding of students, we see patterns that confirm the validity and reliability of the methodological approach. For example, drawings with teachers that are larger than the depicted students also show other signs of teacher-centered learning environments; and with regard to classes, we see patterns regarding learner- or teacher-centeredness that matched with the observational notes taken in those classes.

Whilst a sample of 104 collected drawings is relatively an ideal sample size, it is worth noting that a *limitation* of this study is its sample and sample size. It would be favorable to have a larger and importantly more diverse sample (e.g., students of different grades from different schools; or even students from different countries). Researchers in this field are encouraged to further explore the possibilities of drawing tasks for assessing beliefs and experiences with larger and diverse groups.

## Data availability statement

The raw data supporting the conclusions of this article will be made available by the authors, without undue reservation.

## Ethics statement

The studies involving human participants were reviewed and approved by University of Cologne. Written informed consent to participate in this study was provided by the participants’ legal guardian/next of kin.

## Author contributions

BR did most of the data analyses (including the development of the coding manual, together with LB) and manuscript writing (especially the Methodology, Results, and Discussion sections). LB gathered the data and did most of the data analyses (including the development of the coding manual, together with BR). VH helped with writing the manuscript, especially the Theoretical Background section. All authors contributed to the article and approved the submitted version.

## Funding

We acknowledge support for the Article Processing Charge from the DFG (German Research Foundation, 491454339).

## Conflict of interest

The authors declare that the research was conducted in the absence of any commercial or financial relationships that could be construed as a potential conflict of interest.

## Publisher’s note

All claims expressed in this article are solely those of the authors and do not necessarily represent those of their affiliated organizations, or those of the publisher, the editors and the reviewers. Any product that may be evaluated in this article, or claim that may be made by its manufacturer, is not guaranteed or endorsed by the publisher.

## Footnotes

^1^The students were asked to “Draw your mathematics lessons with your teacher,” drawing students was not required; however, it is hard to imagine a teacher working without students.
