# Supplementary material for: What do mathematics lessons look like? Analyses of primary students’ drawings
Source: Front Psychol. 2023 Jul 13;14:1019299. doi: 10.3389/fpsyg.2023.1019299 (PMC10374451; doi:10.3389/fpsyg.2023.1019299)
Supplement: Supplementary file 1 [file Data_Sheet_1.pdf]

Appendix A

The drawing task

(1) Draw your mathematics lessons with your teacher. The picture should show what you know about the teacher’s work.

Appendix B: Coding Manual

Cat.	Codes	Description
Teacher	Position page<br>Location<br>Classroom<br>Position to students<br>Relative size<br>Teacher activity	Is a teacher present?<br>• No<br>• Yes, drawn.<br>• Yes, written.<br>• Yes, drawn and written.<br>What is the teacher's position on the page?<br>• The page is divided into 3x3 = 9 quadrats (1-3 in the top row, 4-6 in the middle row, and 7-9 in the bottom row); mark all quadrants in which the teacher is present (especially in comic strips)<br>• N/A (Not applicable) if there is no teacher.<br>What is the teachers' location in the classroom?<br>• Near board<br>• At teacher's desk<br>• Near students' desks<br>• Various (in comic strips)<br>• N/A if there is no teacher.<br>What is the teacher's position regarding the students?<br>• Near students<br>• Distant to students<br>• Uncertain<br>• N/A if there are no teachers and/or no students.<br>What is the relative size of the teacher compared to the students (considering teachers are adults and students are children, especially at primary schools)?<br>• Smaller [than students]<br>• Same [about the same size as students]<br>• Bigger [than students]<br>• N/A if no teacher or students are depicted.<br>What is the teacher's (observable) activity? [multiples can be coded]<br>• None<br>• Pointing (to the board)<br>• Writing or drawing on the board<br>• Handing out materials<br>• Writing (if there is chalk or a pen in the teacher's hand and it is used to write something)<br>• Speaking (including lecturing or dictating, see conversation code)<br>• Thinking (for teacher activity)<br>• N/A if not a teacher is depicted.

Students	Quantity<br>Position<br>Activity	Are students present?<br>• No<br>• Yes, drawn.<br>• Yes, written.<br>• Yes, drawn and written.<br>What is the students' (observable) quantity?<br>• None<br>• One<br>• Two<br>• Three or four<br>• More than four<br>What is/are the students' position/s?<br>• Students at their place<br>• Students not at their desks (standing or sitting in the classroom) – middle of the classroom<br>• Students not at their desks – near board / near teacher's desk<br>• N/A if not a student is depicted.<br>What is/are the students' activity/activities?<br>• Working alone [if there is a thinking or speech bubble with a task]<br>• Working in pairs<br>• Working in groups<br>• Other<br>• N/A if undefined.
Teacher-student conversation	Content teacher<br>Content student	Is there a conversation?<br>• None<br>• Only teacher speaks.<br>• Only students speak.<br>• Teacher and students speak.<br>• N/A if there are neither a teacher nor students.<br>What is the content of the teacher conversation? [multiples can be coded]<br>• None (if there is conversation, but the teacher does not speak)<br>• Asking students to answer a question.<br>• Dictates or gives a task verbally.<br>• Lectures or disciplines.<br>• Invites discussion (e.g., praises, questions).<br>• Gives feedback.<br>• Invites students to take materials.<br>• Announces a worksheet.<br>• Announces a new topic.<br>What is the content of the students' conversation? [multiples can be coded]<br>• None (if there is conversation, but the students do not speak)<br>• Raising a hand or calling "here" or "me" to report to the teacher.<br>• Giving the answer to a teacher's question.<br>• Showing signs of being confused.<br>• Disappointment.<br>• N/A if there is no conversation.
Mathematical content	Mathematical work<br>Representational form	What mathematical (sub-)area is taught [if this can be seen in the form of tasks]? [multiples can be coded]<br>• None<br>• Arithmetic<br>• Geometric drawings or constructions<br>• Length<br>• Other activity<br>What representational forms are depicted? [multiples can be coded]<br>• Enactive<br>• Iconic<br>• Symbolic<br>• Written

Classroom	Student desks<br>Board<br>Technology classroom<br>Tools classroom<br>Technology students<br>Tools student	Are students' desks depicted? How?<br>• None<br>• Single<br>• Rows<br>• Clustered.<br>Is there a board to write on?<br>• None<br>• Black- or whiteboard (most often undistinguishable)<br>• Smartboard (with technology)<br>Are non-technological tools depicted in the classroom (for the teacher)?<br>• None<br>• Pointing stick (for the teacher)<br>• Compass and ruler for the blackboard<br>• Mathematical "toys" for display.<br>Are technological tools depicted in the classroom (for the teacher; in addition to the board)?<br>• None<br>• Overhead projector<br>• Computer (for technology in the classroom)<br>Are non-technological tools present with the students?<br>• None<br>• Pen and paper<br>• Compass and ruler for the students<br>• Set square.<br>• Mathematical "toys" for each student.<br>Are technological tools present with the students?<br>• None<br>• Calculators<br>• Other

Appendix C: Supplementary statistical thoughts

In section 4.3.2, we compared frequencies of drawn persons or objects in all pictures with pictures in which the teachers are depicted "bigger" than the students (see Fig. 5 for two examples).

(2) Teacher's position		
Centre	19	18.3 %
Left	25	24.0 %
Right	32	30.8 %
Upper or lower edge	10	9.6 %
Comics	7	6.7 %
No teacher	11	10.6 %
**Sum**	**104**	**100 %**

(2b) Teacher's position		
Centre	13	29.0 %
Left	10	22.2 %
Right	13	29.0 %
Upper or lower edge	4	8.9 %
Comics	2	4.4 %
Other	3	6.7 %
**Sum**	**45**	**100 %**

(3) Students' position		
At their place	38	36.5 %
Not at their place	17	16.4 %
Both (multiple s.)	4	3.9 %
No students depicted	45	43.3 %
**Sum**	**104**	**100 %**

(3b) Students' position		
At their place	24	53.3 %
Not at their place	9	20.0 %
Both (multiple s.)	4	8.9 %
No students[2]	8	17.8 %
**Sum**	**45**	**100 %**

Fig. 5: Comparisons of all pictures with pictures in which the teachers are depicted "bigger"

These observations can be further analyzed with the help of basic inferential statistics. To use chi-square tests, one cannot compare a whole group with a sub-group of itself. Instead, we compare pictures in which teachers are depicted "bigger" (labelled "b") with the remaining pictures (labelled "non-b"). In Tables 11 – 15, now with an additional "c", which stands for comparison, the observed numbers are presented. Additionally, expected numbers (calculated by margin totals) are added in brackets. Cells with a high deviation between these two values are highlighted. Results of the chi-square tests have to be interpreted with caution as some cell values and/or expected cell values are too low. Overall, these analyses confirm the conclusions drawn in section 4.3.2.

**(2c) Teacher's position**			
	**non-b**	**b**	**Sum**
Centre	6 (10.8)	13 (8.2)	**19**
Left	15 (14.2)	10 (10.8)	**25**
Right	19 (18.2)	13 (13.8)	**32**
Upper or lower edge	6 (5.7)	4 (4.3)	**10**
Comics	5 (4.0)	2 (3.0)	**7**
Other	8 (6.2)	3 (4.8)	**11**
**Sum**	**59**	**45**	**104**
$\chi^2 = 6.9$	$p = 0.23$	Yates-$p = 0.45$	

Table 11: Comparison of teacher's position in pictures with "bigger" teachers (b) and the remaining pictures (non-b)

**(3c) Students' position**			
	**non-b**	**b**	**Sum**
At their place	14 (21.6)	24 (16.4)	**38**
Not at their place	8 (9.6)	9 (7.4)	**17**
Both (multiple s.)	0 (2.3)	4 (1.7)	**4**
No students	37 (25.5)	8 (19.5)	**45**
**Sum**	**59**	**45**	**104**
$\chi^2 = 23.9$	$p < 0.001$	Yates-$p < 0.001$	

Table 12: Comparison of students' positions in pictures with "bigger" teachers (b) and the remaining pictures (non-b)

(4c) Students' desks			
	**non-b**	**b**	**Sum**
Single	9 (12.5)	13 (9.5)	**22**
Rows	10 (13.6)	14 (10.4)	**24**
Clustered	4 (5.1)	5 (3.9)	**9**
None	36 (27.8)	13 (21.2)	**49**
**Sum**	**59**	**45**	**104**
$\chi^2 = 10.6$	$p = 0.01$	Yates-$p = 0.04$	

Table 13: Comparison of the positions of students' desks in pictures with "bigger" teachers (b) and the remaining pictures (non-b)

(5c) Students' activities			
	**non-b**	**b**	**Sum**
Working alone	13 (19.9)	22 (15.1)	**35**
w. in group	2 (1.1)	0 (0.9)	**2**
Other	7 (12.5)	15 (9.5)	**22**
No students	37 (25.5)	8 (19.5)	**45**
**Sum**	**59**	**45**	**104**
$\chi^2 = 24.5$	$p < 0.001$	Yates-$p < 0.001$	

Table 14: Comparison of students' activities in pictures with "bigger" teachers (b) and the remaining pictures (non-b)

(6c) Teacher-student conversation			
	**non-b**	**b**	**Sum**
Only teacher	6 (7.9)	8 (6.1)	**14**
Only students	2 (2.8)	3 (2.2)	**5**
Teacher and students	8 (9.1)	8 (6.9)	**16**
No conversation	40 (37.4)	26 (28.6)	**66**
Not applicable	3 (1.7)	0 (1.3)	**3**
**Sum**	**59**	**45**	**104**
$\chi^2 = 4.7$	$p = 0.32$	Yates-$p = 0.75$	

Table 15: Comparison of teacher-student conversation in pictures with "bigger" teachers (b) and the remaining pictures (non-b)
